# Effect of cognitive reserve on cognitive performance in Subjective Cognitive Decline: insights from the BRASCODE Cohort

**DOI:** 10.1002/alz70857_106150

**Published:** 2025-12-26

**Authors:** Manuella Edler Zandoná Giordani, Barbara Loeblein Uebel, Victória Tizeli Souza, Simone Sieben da Mota, Bruno De Oliveira De Marchi, Carolina Rodrigues Formoso, Gabriela Raquel Paz Rivas, Guilherme Da Silva Carvalho, Haniel Bispo De Souza, Lucas Bastos Beltrami, Rhaná Carolina Santos, Samuel Masao Suwa, Wesley Slaviero, Ana Letícia Amorim de Albuquerque, Matheus Zschornack Strelow, Wyllians Vendramini Borelli, Giovanna Carello‐Collar, Maila Rossato Holz, Marcia L. Fagundes Chaves, Eduardo R. Zimmer, Raphael Machado Castilhos

**Affiliations:** ^1^ Universidade Federal do Rio Grande do Sul, Porto Alegre, RS, Brazil; ^2^ Hospital de Clínicas de Porto Alegre, Porto Alegre, RS, Brazil; ^3^ Hospital de Clínicas de Porto Alegre, Porto Alegre, Rio Grande do Sul, Brazil; ^4^ Universidade Federal do Rio Grande do Sul, Porto Alegre, Brazil; ^5^ Universidade do Vale do Rio dos Sinos, São Leopoldo, Rio Grande do Sul, Brazil; ^6^ Clinical Hospital of Porto Alegre, Porto Alegre, Rio Grande do Sul, Brazil; ^7^ PUCRS, Porto Alegre, Rio Grande do Sul, Brazil

## Abstract

**Background:**

Cognitive reserve refers to the brain's capacity to maintain performance despite pathology and may influence the risk of cognitive decline. Therefore, studying the effect of cognitive reserve on older adults with memory complaints can provide insights into their cognitive trajectories and lifestyle factors that could impact on their cognitive deterioration.

**Methods:**

BRASCODE is an observational, prospective cohort study of cognitively unimpaired individuals with > 65 years‐old with cognitive complaints conducted at the Hospital de Clínicas de Porto Alegre, Brazil. The 1‐year follow‐up assessment began in March 2023 and is still ongoing. Sociodemographic data, Memory Complaint Scale (MCS), Mini‐Mental State Examination (MMSE), Clinical Dementia Rating (CDR) Scale and Cognitive Reserve Scale (CRS) were collected. In addition to the cognitive battery, patients were also assessed for behavioral and sleep symptoms using the Neuropsychiatric Inventory (NPI), the Mild Behavioral Impairment Checklist (MBI‐c), the Geriatric Anxiety Inventory (GAI), the Geriatric Depression Scale (GDS) and the Pittsburgh Sleep Quality Index (PSQI).

**Results:**

To date, 100 patients have completed their 1‐year assessment, with 75% being women. The median age was 71 years, 87% of participants were classified as having Subjective Cognitive Decline (SCD), and 28% as having low cognitive reserve (CR). The PSQI correlated positively with GAI scores (rho=0.254, *p* = 0.254). The subject MBI‐c correlated with global cognition (rho=0.225, *p* = 0.0273), and the informant MBI‐c correlated with the CDR‐sum of boxes (rho=0.282, *p* = 0.007). Compared to participants with high CR, those with low CR were more likely to be older, have fewer years of education, be male, and display significantly lower scores on global cognitive assessments (CDR and MMSE). In mixed linear regression, cognitive reserve did not significantly predict global cognition composite score (*p* = 0.419). However, education was a significant predictor (beta = ‐0.009; *p* = 0,0106).

**Conclusion:**

Our results reinforce the impact of education and neuropsychiatric symptoms on cognitive performance in SCD. Follow‐up data and Alzheimer's Disease (AD) biomarkers analysis will help clarify the role of cognitive reserve on their cognitive trajectories.

